# Multi-layer self-calibrated algorithm for transabdominal fetal pulse oximetry: simulation and *in vivo* validation

**DOI:** 10.1088/2515-7647/ae1a27

**Published:** 2025-11-11

**Authors:** Jingyi Wu, Martin P Debreczeny, Nevan C Hanumara, Neil Ray, Baptiste Jayet, Stefan Andersson-Engels, Jana M Kainerstorfer

**Affiliations:** 1 Department of Biomedical Engineering, Carnegie Mellon University, Pittsburgh, PA, United States of America; 2 Raydiant Oximetry, Inc., San Ramon, CA, United States of America; 3 Department of Mechanical Engineering, Meassachusetts Institute of Technology, Cambridge, MA, United States of America; 4 Biophotonics@Tyndall, Tyndall National Institution, T12 R5CP Cork, Ireland; 5 School of Physics, University College Cork, T12 K8AF Cork, Ireland; 6 Neuroscience Institute, Carnegie Mellon University, Pittsburgh, PA, United States of America

**Keywords:** transabdominal fetal pulse oximetry, near-infrared spectroscopy, multi-layer photon transport, photon partial pathlength, Monte Carlo simulation

## Abstract

Transabdominal fetal pulse oximetry offers a promising approach to non-invasively monitor fetal arterial oxygen saturation (SaO_2_), potentially enhancing clinical decision-making and reducing unnecessary interventions during delivery. However, accurate estimation of fetal SaO_2_ (denoted as SpO_2_ when measured non-invasively) is complicated by the multi-layer maternal-fetal tissue structure, distinct maternal and fetal physiological signals, and inherently low fetal oxygen saturation levels. A multi-layer self-calibrated algorithm was developed by combining the multi-layer modified Beer–Lambert law with an analytical photon partial pathlength model. This approach distinguishes maternal and fetal tissue contributions, enabling more accurate fetal SpO_2_ estimation. Validation was performed using Monte Carlo photon simulations of multi-layer tissue geometries, where synthetic optical signals representing fetal cardiac pulsations were generated under two fetal depths and randomly varied maternal and fetal oxygen saturations and optical properties. Further validation was performed using *in vivo* sheep data, where fetal SpO_2_ values derived from transabdominal continuous-wave near-infrared spectroscopy measurements were compared against reference fetal SaO_2_ from CO-oximetry. In simulations, the algorithm achieved a mean absolute error (MAE) below 5% and a Pearson correlation coefficient (*R*) of 0.98 between estimated fetal SpO_2_ and ground truth fetal SaO_2_ when using optimal input parameters. In the sheep experiment, agreement with reference measurements was maintained (MAE = 10.3%, *R* = 0.91). However, algorithm performance was highly sensitive to accurate optical properties and tissue layer thicknesses inputs, which may be challenging to obtain in clinical settings. These results demonstrate proof-of-concept feasibility for the multi-layer self-calibrated algorithm in both simulated and *in vivo* conditions. While further refinement, particularly in optical property estimation and fetal depths in human pregnancies, is necessary, this work provides a foundational framework for the future clinical translation of non-invasive fetal SpO_2_ monitoring.

## 1 Introduction

Fetal oxygenation is critical for maintaining fetal well-being throughout pregnancy and delivery. During labor, maternal contractions can compress uterine and umbilical vessels, reducing oxygen delivery to the fetus. This condition, known as fetal hypoxia, can cause severe complications, including neurological impairment and, in extreme cases, fetal demise [1]. Despite its clinical significance, there currently exists no direct, non-invasive method for measuring fetal oxygenation.

Current fetal monitoring during delivery primarily relies on cardiotocography (CTG), which tracks fetal heart rate (HR) and maternal contractions. Because fetal HR is influenced by autonomic regulation and fetal oxygenation, abnormal fetal HR patterns are used as indicators of possible intrapartum fetal hypoxia [2]. However, fetal HR interpretation is subjective and prone to error. CTG thus has a low predictive rate of ∼30% and a high false-positive rate of ∼60% for predicting fetal hypoxia, meaning many abnormal CTG traces do not correspond to actual fetal hypoxia [3–5]. Several alternative modalities have been investigated to overcome CTG’s limitations. Abdominal fetal electrocardiography (afECG) detects the fetal cardiac electrical signal through surface electrodes and provides higher-quality HR tracings and beat-to-beat variability assessment compared to CTG [6, 7]. However, afECG does not measure oxygenation and could be affected by maternal ECG contamination, electrical impedance from maternal adipose, and fetal position. Fetal blood sampling can directly quantify blood gases but is semi-invasive, feasible only after membrane rupture, and unsuitable for continuous monitoring [8, 9]. Transvaginal fetal pulse oximetry provides direct fetal oxygen saturation but is invasive and clinically impractical, leading to the withdrawal of early commercial devices [10, 11]. These limitations highlight the need for a direct and non-invasive method for tracking fetal oxygenation in real time.

Generally, arterial oxygen saturation (SaO_2_) is most accurately determined by arterial blood sampling and blood gas analysis. However, in most clinical and home environments, arterial blood sampling is impractical, so SaO_2_ is estimated non-invasively as SpO_2_ using pulse oximetry [12, 13]. Commercial pulse oximeters operate in both transmission (e.g. finger, ear) and reflectance (e.g. forehead, skin surfaces) modes, relying on photoplethysmographic detection of pulsatile absorption changes of the arterial blood. Regardless of configuration, these devices require empirical calibration on healthy adults undergoing controlled desaturation. As a result, their accuracy is highest within the calibrated range and degrades substantially below ∼80% SpO_2_ [14, 15]. A human fetal‐specific empirical calibration is not feasible because fetal saturations are typically 30%–70%, and ethical and practical constraints preclude collection of human fetal reference SaO_2_ across the required range [16, 17]. Prior transvaginal fetal oximetry placed reflectance sensors on the presenting part (fetal head or cheek) via the vaginal canal and was calibrated in piglets, but clinical adoption was limited by invasiveness and mixed performance [10, 11].

To address the challenge posed by low fetal saturation, a previously described self-calibrated algorithm that leverages the dependence of photon pathlength on SaO_2_ can be incorporated [18]. This method builds upon the modified Beer–Lambert law (mBLL) by taking into account of how pathlength varies with absorption and, hence, SaO_2_. It contrasts with conventional pulse oximetry, which assumes a fixed pathlength ratio and therefore requires empirical calibration [19, 20]. The algorithm estimates SaO_2_ by fitting the measured change in optical density from the cardiac pulsation to the mBLL, using a saturation-dependent analytical pathlength. This approach was validated in cerebral measurements from breath‐holding freedivers, where SaO_2_ decreased to as low as ∼41%. These results demonstrated accurate SaO_2_ estimation across a wide saturation range, supporting the relevance of this method for fetal pulse oximetry, which inherently involes low SaO_2_ levels.

Transabdominal fetal pulse oximetry, however, faces additional challenges. Light needs to travel through multiple maternal layers (adipose, muscle, uterus) before reaching the fetal tissue and returning to the detector in a reflectance mode. Also, maternal and fetal compartments have distinct optical properties, hemoglobin concentrations, oxygen saturations, thicknesses, and HRs [16, 21, 22]. Thus, the measured reflectance signal on the maternal abdomen is a multi‐parameter function of layer‐specific information, containing overlapping maternal and fetal pulsatile components, with the fetal contribution typically a small fraction of the signal due to its greater depth (∼3 cm versus ∼1 cm for adult cerebral cortex) [21, 22]. These factors render single‐layer models inadequate and motivate the development of a multi‐layer formulation that can properly attribute absorption changes to fetal tissue.

Recognizing these complexities, this study extends the previously reported self‐calibrated framework into a multi‐layer model that incorporates analytical partial pathlength calculations for each tissue layer. After separating maternal and fetal pulsatile components, this model attributes absorption changes to the appropriate layer and estimates fetal SaO_2_ without empirical calibration. We validate this approach first with Monte Carlo simulations spanning physiologically plausible optical properties, depths, and source–detector (SD) separations, and then with an *in‐vivo* sheep experiment, comparing algorithm‐derived fetal SpO_2_ against ground truth (GT) SaO_2_ measurements from CO-oximetry.

As a proof‐of‐concept, our results demonstrate that the multi‐layer self‐calibrated algorithm can effectively track fetal SpO_2_ dynamics under both simulated and experimental conditions. To our knowledge, this study represents the first implementation of a layer-resolved, self-calibrated algorithm for transabdominal fetal pulse oximetry, validated using both Monte Carlo photon simulation and *in vivo* sheep experiment. However, algorithm performance remains dependent on reasonable estimates of layer optical properties and thickness, and the sheep model involved shallower fetal depths than are typical in human pregnancies. Within these constraints, this work establishes a physics‐based foundation toward non‐invasive transabdominal fetal SpO_2_ monitoring.

## 2 Method

### 2.1 Overview of the self-calibrated pulse oximetry algorithm assuming tissue homogeneity

The self-calibrated pulse oximetry algorithm previously reported leverages the inherent dependency of the photon mean pathlength (〈*L*〉) on the tissue absorption coefficient (${\mu _{\text{a}}}$), which depends on arterial oxygen saturation.

According to the mBLL, the wavelength (*λ*) dependent pulsatile optical density change (ΔOD) due to cardiac-induced arterial blood volume variations at the HR frequency is described as [19, 20]:

\begin{equation*}\begin{array}{*{20}{c}} {\Delta {\text{OD}}_{{\text{analytical}}}^{{\lambda _n}} = \ln \left( {\frac{{I_{\text{d}}^{{\lambda _n}}}}{{I_{\text{s}}^{{\lambda _n}}}}} \right) = {{\langle L\rangle }^{{\lambda _n}}}\Delta \mu _{\text{a}}^{{\lambda _n}},} \end{array}\end{equation*}

where *n* = 1, 2, 3, … represents measurements at multiple wavelengths, *I*_d_ and *I*_s_ are the measured light intensities at diastole and systole, respectively, and Δ${\mu _{\text{a}}}$ is the pulsatile change in the absorption coefficient due to arterial hemoglobin concentration changes.

To enable fitting for SpO_2_, both 〈*L*〉 and ${{\Delta }}{\mu _{\text{a}}}$ are expressed explicitly as functions of SpO_2_. First, the photon mean pathlength 〈*L*〉 for reflectance geometry in a semi-infinite medium is analytically given by [23]:

\begin{equation*}\begin{array}{*{20}{c}} {{{\langle L\rangle }^{{\lambda _n}}} = \frac{{\sqrt {3\mu {^{^{\prime}}}_{\text{s}}^{{\lambda _n}}} }}{{2\sqrt {\mu _{\text{a}}^{{\lambda _n}}} }}\frac{{{r^2}\sqrt {3\mu _{\text{a}}^{{\lambda _n}}\mu {^{^{\prime}}}_{\text{s}}^{{\lambda _n}}} }}{{r\sqrt {3\mu _{\text{a}}^{{\lambda _n}}\mu {^{^{\prime}}}_{\text{s}}^{{\lambda _n}}} + 1}}} \end{array},\end{equation*}

where *r* is the SD distance, and $\mu _{\text{s}}^{\prime}$ is the reduced scattering coefficient, typically obtained from literature or assumed based on tissue type.

Next, to incorporate the SpO_2_ dependence, ${\mu _{\text{a}}}$ in equation (2) is expressed in terms of SpO_2_ and the total hemoglobin concentration [HbT]. Considering only arterial blood, with [HbT] = [HbO] + [Hb] and SpO_2_ = [HbO]/[HbT], ${\mu _{\text{a}}}$ can be expressed by:

\begin{equation*}\begin{array}{*{20}{c}} {\mu _{\text{a}}^{{\lambda _n}} = \left[ {{\text{HbO}}} \right]\varepsilon _{{\text{HbO}}}^{{\lambda _n}} + \left[ {{\text{Hb}}} \right]\varepsilon _{{\text{Hb}}}^{{\lambda _n}}} \\ { = \left[ {\varepsilon _{{\text{HbO}}}^{{\lambda _n}}{\text{Sp}}{{\text{O}}_2} + \varepsilon _{{\text{Hb}}}^{{\lambda _n}}\left( {1 - {\text{Sp}}{{\text{O}}_2}} \right)} \right]\left[ {{\text{HbT}}} \right],{ }} \end{array}\end{equation*}

where [HbO] and [Hb] are the concentration of oxy- and deoxy-hemoglbin, respectively, $\varepsilon $ denotes the extinction coefficient. Here, [HbT] needs to be set as a physiologically reasonable constant (e.g. 50 *μ*M).

Similarly, the pulsatile absorption change ${{\Delta }}{\mu _{\text{a}}}$ during cardiac pulsation can be defined by considering small fractional variations in [HbT], so similar to equation (3):

\begin{equation*}\begin{array}{*{20}{c}} {\Delta \mu _{\text{a}}^{{\lambda _n}} = \left[ {\varepsilon _{{\text{HbO}}}^{{\lambda _n}}{\text{Sp}}{{\text{O}}_2} + \varepsilon _{{\text{Hb}}}^{{\lambda _n}}\left( {1 - {\text{Sp}}{{\text{O}}_2}} \right)} \right]\Delta \left[ {{\text{HbT}}} \right],{ }} \end{array}\end{equation*}

where Δ[HbT] represents a small pulsatile change in [HbT], which is assumed as a small fixed percentage (e.g. 5%) of the baseline [HbT].

Before fitting for SpO_2_, ΔOD across wavelengths need to be normalized at a reference wavelength (usually the first wavelength, *λ*_1_). This normalization mitigates uncertainties arising from assumptions of constant parameters and highlights the wavelength-dependent spectral shape solely influenced by SpO_2_. The normalized measured and analytical ΔOD are thus given by:

\begin{equation*}\begin{array}{*{20}{c}} \widehat {{\Delta {\text{OD}}}}_{{\text{measured}}}^{{\lambda _n}} = {\Delta {\text{OD}}}_{{\text{measured}}}^{{\lambda _n}}/{{\Delta {\text{OD}}}_{{\text{measured}}}^{{\lambda _1}},} \end{array}\end{equation*}

\begin{equation*}\begin{array}{*{20}{c}} \widehat {{\Delta {\text{OD}}}}_{{\text{analytical}}}^{{\lambda _n}} = {\Delta {\text{OD}}}_{{\text{analytical}}}^{{\lambda _n}}/{{\Delta {\text{OD}}}_{{\text{analytical}}}^{{\lambda _1}}} \end{array}.\end{equation*}

Finally, SpO_2_ can be determined by minimizing the residual sum-of-squares (RSSs) between the normalized and measured analytical ΔOD spectra:

\begin{equation*}\begin{array}{*{20}{c}} {\text{RSS}}\left( {\text{Sp}{\text{O}}_2} \right) = \mathop \sum \limits_{n = 1}^N \left[ {\widehat {\Delta {\text{OD}}}}_{\text{measured}}^{\lambda _n} - \widehat {\Delta {\text{OD}}}_{\text{analytical}}^{{\lambda _n}}\left( {\text{Sp}}{\text{O}}_2 \right) \right]^2 \end{array}.\end{equation*}

In practice, this optimization typically involves a straightforward one-dimensional grid search with a defined resolution (e.g. 1% increments across 0%–100% SpO_2_ range). It is computationally efficient and robust due to the simplicity of the search space.

This self-calibrated approach has shown to be applicable for cerebral oximetry applications, including breath-holding freediver experiments in which SpO_2_ levels dropped to as low as 41%. The algorithm demonstrated robust performance across a broad SpO_2_ range, accurately tracking desaturation events [18].

### 2.2 Extension of the self-calibrated algorithm to multi-layer tissue

The previously described self-calibrated algorithm assumes a homogeneous tissue medium and does not account for absorption changes occurring in different layers. While this simplification can be adequate for shallow tissues such as the brain or more homogeneous sites like the arm and finger, it is insufficient for transabdominal fetal pulse oximetry, where fetal tissues lie deep beneath multiple maternal layers, and maternal and fetal tissues exhibit distinct SaO_2_ values, hemoglobin concentrations, and HRs.

To address this limitation for transabdominal fetal pulse oximetry, we extend the algorithm to multi-layer tissue by incorporating photon partial pathlengths, which describes the fraction of the total photon path spent in each tissue layer, allowing separate attribution of absorption changes to different layers [24–27]. The single-layer mBLL is generalized to the multi-layer form [24, 26]:

\begin{equation*}\begin{array}{*{20}{c}} {\Delta {\text{OD}} = {{\Sigma }}_{i = 1}^n\langle {L_i}\rangle {{\Delta }}{\mu _{{\text{a}},i}},{ }} \end{array}\end{equation*}

where *n* is the number of layers, $\langle {L_i}\rangle $ is the partial pathlength in the *i*th layer, and ${{\Delta }}{\mu _{{\text{a}},i}}$ is the corresponding pulsatile absorption change in that layer.

In a simplified form, for transabdominal fetal pulse oximetry specifically, the measured optical density change from the maternal abdomen (ΔOD_measured_) contains both maternal and fetal contributions:

\begin{equation*}\begin{array}{*{20}{c}} {{{\Delta }}{{\text{OD}}_{{\text{measured}}}} = \langle {L_{{\text{mother}}}}\rangle {{\Delta }}{\mu _{{\text{a, mother}}}} + \langle {L_{{\text{fetus}}}}\rangle {{\Delta }}{\mu _{{\text{a,fetus}}}}{ }} \end{array}.\end{equation*}

Since fetal HR is typically higher than maternal HR [16], frequency-domain filtering methods, such as spectrogram-based extraction, can isolate the fetal signal by identifying optical density changes occurring at the fetal HR frequency. Thus, the isolated fetal component becomes:

\begin{equation*}\begin{array}{*{20}{c}} {{{\Delta }}{{\text{OD}}_{{\text{fetus}}}} = \langle {L_{{\text{fetus}}}}\rangle {{\Delta }}{\mu _{{\text{a,fetus}}}}{ }} \end{array}.\end{equation*}

The critical unknown here is the fetal partial pathlength, $\langle {L_{{\text{fetus}}}}\rangle $, which must be determined for the multi-layer self-calibrated algorithm to estimate fetal SpO_2_.

Studies have derived analytical expressions for photon partial pathlengths in multi-layered cylindrical tissue media, offering a computationally efficient approach to estimating layer-specific partial pathlengths [24, 28, 29], which are calculated using the analytical expression for diffuse reflectance (*R*) as:

\begin{equation*}\begin{array}{*{20}{c}} {\langle {L_i}\rangle \left( r \right) = \frac{{ - 1}}{{R\left( r \right)}}\frac{{\partial R\left( r \right)}}{{\partial {\mu _{{\text{a,}}i}}}}{ }} \end{array}.\end{equation*}

This analytical expression has been validated against Monte Carlo simulations in simplified multi-layer geometries. Although it assumes uniform layer thicknesses, homogeneous optical properties within each layer, and relatively simple anatomical shapes, it nonetheless provide a reasonable approximation for modeling photon propagation in multi-layer biological tissue [24, 30, 31], and it is suitable for our proof-of-concept development.

By integrating analytical partial-pathlength calculations into the original self-calibrated algorithm, we generalize the model from a homogeneous to a multi-layer tissue representation. The fundamental principle remains the same: fitting the isolated ΔOD_fetus_ spectrum to the mBLL with a saturation‐dependent analytical partial pathlength. This extension allows us to estimate fetal SpO_2_ in the multi-layered environment characteristic of transabdominal fetal pulse oximetry, addressing key limitations of previous single-layer assumptions.

### 2.3 Validation with Monte Carlo simulation

To validate the multi-layer self-calibrated algorithm, we generated synthetic data using Monte Carlo simulations in MCXLAB [32]. We systematically controlled tissue optical properties, maternal and fetal SpO_2_, and layer thickness to evaluate the algorithm under different conditions.

#### 2.3.1 Multi-layer tissue model

A three-layer slab geometry was used to represent the main optical compartments encountered in transabdominal fetal pulse oximetry in humans: maternal adipose (layer 1), combined rectus muscle + uterus (layer 2), and fetal tissue (layer 3). Superficial structures such as skin and the amniotic-membrane/fluid layers were omitted because their small thicknesses. This simplified three-layer model provides sufficient anatomical realism to test our algorithm, which is appropriate for the proof-of-concept validation presented here. It should be noted that this three-layer configuration approximates the human abdominal geometry, whereas the *in vivo* sheep model (see section 2.4.3) was simplified to two layers due to shallower fetal depth (∼1.5 cm) and the maternal tissue being not easily separable into distinct optical layers [33].

Fetal depth, defined as the combined thickness of maternal layers above fetal tissue, typically ranges between 1.5 cm and 4 cm in late pregnancy, depending on parameters such as maternal BMI, fetal orientation, stage of labor [16, 34, 35]. We selected two fetal depths—20 mm (shallower) and 35 mm (deeper)—to exemplify clinical variations. Given that muscle and uterine wall thicknesses vary relatively little at term [36, 37], we fixed the combined muscle and uterus thickness at 15 mm and adjusted maternal adipose thickness accordingly (5 mm adipose for 20 mm depth, 20 mm adipose for 35 mm depth).

**Figure 1.**
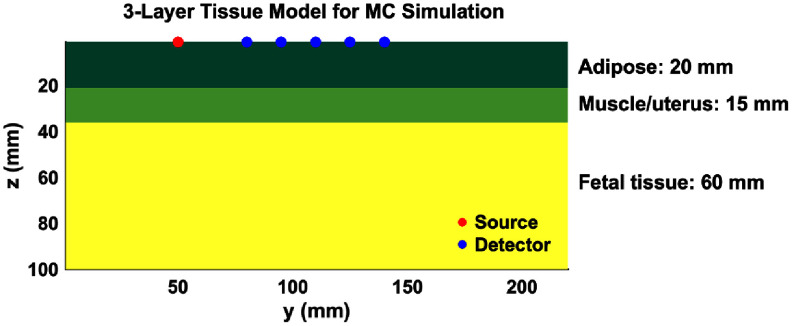
Example 3-layer tissue model with 35 mm fetal depth.

In MCXLAB, a rectangular geometry of 140 × 220 × 100 mm with 1 mm voxels was constructed (figure 1). This ensured sufficient volume to accommodate long SD distances without edge effects. Each simulation involved 3 × 10^9^ photons.

#### 2.3.2 Optical property selection and variability

We simulated 50 virtual subjects for each fetal depth, keeping constant layer thicknesses while introducing inter-subject variability in optical properties. Absorption (${\mu _{\text{a}}}$) and scattering ($\mu _{\text{s}}^{\prime}$) of each layer were randomly sampled within physiologically realistic ranges derived from literature [38–40].

SaO_2_ values for each layer were randomized within clinically plausible ranges. Maternal SaO_2_ was randomly set between 90%–100%, identical in both maternal layers. Fetal SaO_2_ ranged from 30%–70%.

Six wavelengths (700, 730, 760, 800, 830, 860 nm) spanning the NIR region were simulated, capturing key absorption spectral features of oxy- and deoxyhemoglobin. For each layer, the absorption coefficient was calculated from hemoglobin concentrations and SaO_2_ values by. Note that the hardware device used in the *in vivo* validation (see section 2.4.1) operates at different wavelengths (756–855 nm) but covers a similar spectral range and features. Small differences near key spectral regions are not expected to affect the self-calibrated algorithm, which relies on the relative spectral shape and ratio-based features of ΔOD across wavelengths rather than absolute intensity values.

Fetal arterial pulsations were modeled by a 5% increase in ${\mu _{\text{a}}}$ between diastolic (baseline) and systolic states in the fetal layer, while maternal layer absorption remained constant.

The reduced scattering coefficient for each layer was modeled using a power-law decay across wavelength, $\mu _{\text{s}}^{^{^{\prime}}} = a{\left( {\lambda /{\lambda _0}} \right)^{ - b}}$, where $a$ (scattering amplitude, unit mm^−1^) and $b$ (scattering power, unitless) were randomized within literature-based ranges, and *λ*_0_ is the reference wavelength (800 nm in this case) [38].

**Figure 2.**
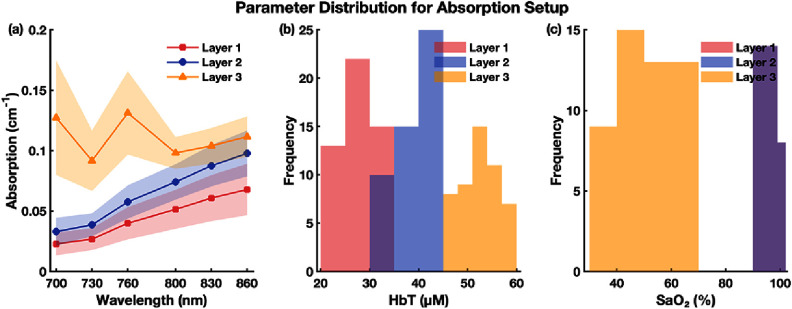
Distributions of ${\mu _{\text{a}}}$, HbT, and SaO_2_ for the 3-layer tissue model. (a) Spectra across 700–860 nm for each layer. (b) Distribution of HbT across virtual subjects per layer. (c) Distribution of SaO_2_ for each layer. The bars for layer 1 and layer 2 are overlapped because maternal layers were set to have the same SaO_2_.

**Figure 3.**
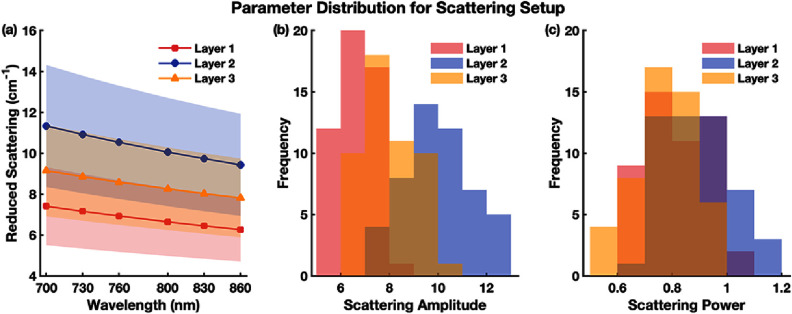
Distributions of $\mu _{\text{s}}^{\prime}$, scattering amplitude, and power, for the 3-layer tissue model. (a) $\mu _{\text{s}}^{\prime}$ spectra across 700–860 nm for each layer. (b) Distribution of scattering amplitude across virtual subjects per layer. (c) Distribution of scattering power across virtual subjects per layer.

The distributions and variabilities of these optical parameters are summarized in figures 2 and 3, with the mean (solid line) and two standard deviations (shaded region).

#### 2.3.3 Fetal signal extraction

Each virtual subject underwent two simulations: a diastolic phase (baseline fetal ${\mu _{\text{a}}}$) and a systolic phase (fetal ${\mu _{\text{a}}}$ increased by 5%). The simulated fetal pulsatile signal (ΔOD_fetus_) was computed as:

\begin{equation*}\begin{array}{*{20}{c}} {{{\Delta}}{{\text{OD}}_{{\text{fetus}}}} = \ln \frac{{{I_{{\text{fetus}},{\text{ diastole}}}}}}{{{I_{{\text{fetus}},{\text{ systole}}}}}},} \end{array}\end{equation*}

where *I*_fetus_ is the simulated detected intensity at the maternal surface for each cardiac phase.

To enhance detectability of the weak fetal signal, particularly at larger SD distances, we modeled detectors with large area (10 × 10 mm) aiming to capture a greater fraction of photons returning from the deep fetal layer [40, 41]. Surface ΔOD_fetus_ values on the 1 mm simulation grid were first interpolated onto a finer grid (<0.1 mm resolution), and then spatially averaged within the detector area. Detectors were placed at SD distances from 30 mm to 90 mm.

#### 2.3.4 Evaluation of algorithm robustness to input uncertainties

Extracted fetal signals (ΔOD_fetus_) across all wavelengths and SD distances were input into the multi-layer self-calibrated algorithm to estimate fetal SpO_2_. To evaluate algorithm’s robustness, we performed perturbation analysis to quantify algorithm sensitivity to uncertainties in input parameters, simulating potential scenarios when precise optical and anatomical parameters are hard to estimate. Seven conditions were tested:

- Ideal case: all inputs are true simulation values.
- Optical property perturbations: uniformly increased or decreased layer ${\mu _{\text{a}}}$ or $\mu _{\text{s}}^{\prime}$ by ± 20%.
- Thickness perturbations: uniformly increased or decreased layer thicknesses by ± 20%.

In this paper, we focused on evaluations at two representative SD distances (60 mm and 90 mm), which were picked to include the range of clinical interest, for both fetal depths (20 mm and 35 mm). The shorter SD distance (60 mm) was expected to have limited depth sensitivity, whereas the longer SD distance (90 mm), though more susceptible to photon attenuation, was anticipated to provide better fetal sensitivity.

Algorithm performance was evaluated quantitatively and visually by the following methods: Mean absolute error (${\text{MAE}} = \frac{1}{N}{{\Sigma }}_{n = 1}^N\left| {{\text{Sp}}{{\text{O}}_2} - {\text{Sa}}{{\text{O}}_2}} \right|$) were presented as bar plots, showing quantitative comparisons between estimation and GT. Box plots were used to show the distribution of estimation errors across subjects. Finally, scatter plots of estimation (SpO_2_) versus GT (SaO_2_) and Pearson correlation (*R*) were presented.

### 2.4 Validation with sheep experiment

#### 2.4.1 Experimental design and data collection

Pregnant sheep were chose as the experimental model for our studies because (1) they have singleton pregnancies similar to humans, (2) fetal depths (2–4 cm) are comparable to term human pregnancies, and (3) the fetal hemoglobin oxygen-binding properties closely resemble those of human fetuses [42, 43].

**Figure 4.**
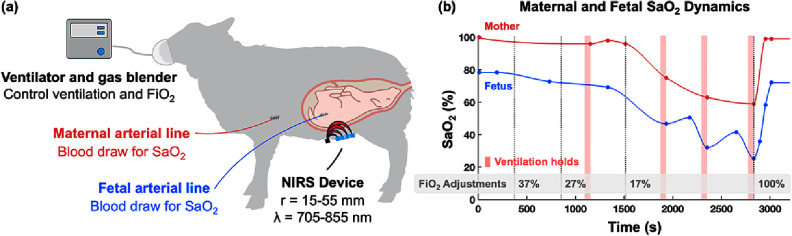
(a) Schematic illustration of the sheep experimental. Maternal and fetal A-lines were used to acquire reference SaO_2_. A transabdominal NIRS probe was positioned over the fetal head (schematically represented). (b) Fetal hypoxia was induced by stepwise reduction of maternal FiO_2_ (vertical dashed lines) and intermittent ventilator holds (shaded intervals), resulting in progressive maternal and fetal desaturation followed by recovery when FiO_2_ was restored to 100%. Illustration of pregnant sheep was created with BioRender.com. Created in BioRender. Wu, J. (2025) [44] https://BioRender.com/p3m5vf3.

The animal study was conducted in collaboration with Raydiant Oximetry Inc. All procedures were approved by the Institutional Animal Care and Use Committee (BioSurg, Inc.; ID: OR0317n) and conducted in accordance with institutional ethical standards and the principles underlying the Declaration of Helsinki. During surgical preparation, pregnant ewes were anesthetized, intubated, and mechanically ventilated. A hysterotomy was performed to partially exteriorize the fetus, keeping the umbilical cord intact. Arterial catheters (A-lines) were inserted into the fetal carotid or femoral artery for blood gas sampling. The fetus was returned into the uterus with catheters securely placed, ensuring fetal proximity (∼15–20 mm) to the maternal abdomen to facilitate fetal signal detection in this proof-of-concept study. Near-infrared spectroscopy (NIRS) measurements were acquired using MetaOx (ISS Inc., Champaign, IL, USA), which has SD distances of 15, 25, 40, and 55 mm, and six near-infrared wavelengths (756, 785, 812, 825, 846, 855 nm) with a 50 Hz sampling rate. Optical probes were positioned on the maternal abdomen directly above the fetal head, and continuous NIRS data were recorded throughout the hypoxia experiments. An illustration of the experiment setup is shown in figure 4(a).

Fetal hypoxia was induced by progressive maternal oxygen desaturation. Maternal inspired oxygen fraction (FiO_2_) was stepwise decreased by a gas blender: from 50% at baseline, to below room air (∼20%), and finally increased to 100% for recovery (figure 4(b), vertical dashed lines). Intermittent 60 s ventilator holds (figure 4(b), shaded interval) were also applied to augment desaturation. The hypoxic period was defined as beginning with the initial FiO_2_ reduction from baseline to 37% close to 500 s, and ending upon restoration to 100% at ∼2800 s. Maternal and fetal arterial blood samples were intermittently collected from the A-lines (figure 4(b), circles) for blood gas analysis via CO-oximetry, providing ground-truth maternal and fetal SaO_2_ values.

#### 2.4.2 Fetal signal extraction from NIRS measurements

To isolate the fetal pulsatile signal (ΔOD_fetus_), fetal HR was identified using the continuous fetal A-line as a reliable physiological reference. Using MATLAB, the raw A-line signal was high-pass filtered at 1 Hz (3rd order zero-phase Butterworth filter) to remove low-frequency noise while preserving pulsatile components. Then, a spectrogram was computed from this filtered signal, from which fetal HR was extracted using the time-frequency ridge (tfridge) method. A 20 s window with 50% overlap was used for the spectrogram to balance temporal and frequency resolution, ensuring accurate capture of distinct maternal and fetal HR components while maintaining sensitivity to short-term physiological variations.

Once the fetal HR time trace was identified, NIRS intensity signals, *I*(*t*), were converted to optical density changes, ${{\Delta O}}{{\text{D}}_{{\text{measured}}}}\left( t \right) = - \ln \left( {I\left( t \right)/{I_0}} \right)$, where *I*_0_ is the mean of *I*(*t*). Spectrograms of ΔOD_measured_ were computed, and the fetal pulsatile signal, ΔOD_fetus_(*t*), was extracted as the spectrogram magnitude at the fetal HR frequency. Figure 10 in section 3.2.1 illustrates this process.

#### 2.4.3 Algorithm evaluation on sheep data

To test the multi-layer self-calibrated algorithm, we selected a high-quality dataset from one representative sheep experiment, in which fetal cardiac pulsations were clearly discernible in the spectrogram (figure 10(b)). Fetal depth was estimated to be approximately 15 mm based on fetal placement during surgery. The longest SD distance available (55 mm) was selected to maximize depth sensitivity.

Considering the anatomical simplicity and minimal adipose thickness in sheep, a two-layer tissue model was used instead of the three-layer configuration used in the simulations [33, 45]. Layer 1 represented maternal tissues (combined adipose, muscle, uterus) with a fixed 15 mm thickness, and layer 2 represented fetal tissue (semi-infinite).

The determination of precise, layer-specific optical properties from NIRS measurements is challenging and beyond the scope of this study [46, 47]. In addition, model misspecification can introduce a systematic offset in the fetal SpO_2_ estimate, as first observed in our simulation results (section 3.1.4). We thus employed a parameter selection strategy based on physiological constraints: A grid search approach was conducted, exploring physiologically plausible ranges of ${\mu _{\text{a}}}$ and $\mu _{\text{s}}^{^{^{\prime}}}$ for both maternal and fetal layers. The objective was to minimize the discrepancy between algorithm-estimated fetal SpO_2_ and blood-gas SaO_2_ within a defined baseline window (before hypoxia induction). The resulting parameter set was then held fixed for all subsequent analysis (hypoxia and recovery).

Reduced scattering coefficients were modeled by the power-law: $\mu _{\text{s}}^{^{^{\prime}}} = a{\left( {\lambda /{\lambda _0}} \right)^{ - b}}$, with scattering power $b$ fixed at 1.0, and the scattering amplitude $a$ (i.e. $\mu _{\text{s}}^{\prime}$ at *λ*_0_ = 800 nm) varied between 0.35 and 1.5 mm^−1^. Same as the simulation study, higher maternal scattering was enforced.

Absorption was approximated as hemoglobin-dominated, with layer-specific absorption calculated as: $\mu _{\text{a}}^{{\lambda _n}} = \left[ {{\text{HbT}}} \right]\left[ {\varepsilon _{{\text{HbO}}}^{{\lambda _n}}{\text{Sa}}{{\text{O}}_2} + \varepsilon _{{\text{Hb}}}^{{\lambda _n}}\left( {1 - {\text{Sa}}{{\text{O}}_2}} \right)} \right]$. Guided by literature and physiological plausibility, maternal HbT ranged from 40–70 *μ*M (i.e. ∼0.007–0.013 mm^−1^ at 800 nm), and fetal HbT from the maternal value up to 100 *μ*M (i.e. ∼0.02 mm^−1^ at 800 nm). Maternal SaO_2_ values were obtained from intermittent arterial blood gas measurements and temporally interpolated to match ΔOD_fetus_(*t*). Fetal SaO_2_ were then estimated by algorithm.

The optimal parameter set from this search, which yielded the closest baseline fetal SpO_2_ alignment to blood gas reference, was HbT = 55 *μ*M and $\mu _{\text{s}}^{\prime}$ (800 nm) = 1.1 mm^−1^ for maternal layer, and HbT = 95 *μ*M and $\mu _{\text{s}}^{\prime}$ (800 nm) = 0.4 mm^−1^ for fetal layer, which are consistent with reported ranges (generally higher maternal scattering and higher fetal absorption) [38–40, 48–50].

Before comparing the estimated fetal SpO_2_ with the GT SaO_2_, we applied a multi-step cleaning and smoothing process in MATLAB. First, values exceeding 95% were replaced with NaN, as these represented algorithm failures that typically yielded capped values near 100%. A Hampel filter (threshold: 2 STD; window length: ∼6 min) was then used to remove outliers. Finally, Gaussian smoothing with the same window length was applied to suppress residual noise while preserving the underlying physiological trends.

Then, to assess algorithm sensitivity to deviations in input ${\mu _{\text{a}}}$ and $\mu _{\text{s}}^{\prime}$, we performed three comparative analyzes:

- Uniform absorption: HbT = 75 *μ*M for both layers (average optimal HbT), scattering kept optimal.
- Uniform scattering: $\mu _{\text{s}}^{\prime}$ (800 nm) = 0.75 mm^−1^ for both layers (average value), absorption kept optimal.
- Uniform absorption and scattering: HbT = 75 *μ*M and $\mu _{\text{s}}^{\prime}$ (800 nm) = 0.75 mm^−1^ for both layers.

These scenarios are relevant for NIRS applications, where subject- and layer-specific optical properties are generally unknown. For each scenario, we evaluated the algorithm’s performance by: (1) visually comparing estimated fetal SpO_2_ time traces against ground-truth fetal SaO_2_ dynamics, and (2) quantifying agreement through scatter plots, Pearson correlation coefficients (*R*), and MAE.

### 2.5 Single-layer and multi-layer algorithm comparison

We compared the previously developed self-calibrated algorithm [18] based on a single-layer assumption (using mean photon pathlength) with the current multi-layer version (using layer-specific partial pathlengths). Both simulated and *in vivo* datasets were analyzed.

For the simulated data, ΔOD_fetus_ values were taken from the three-layer Monte Carlo simulations with fetal depth of 35 mm and SD distance of 90 mm. These values were then used as inputs for both algorithms to estimate fetal SpO_2_. Because the single-layer model does not explicitly account for layered tissue structures, its input optical properties were averaged across all layers for each of the 50 virtual subjects. For the *in vivo* sheep experiments, ΔOD_fetus_ extracted from the 55 mm SD distance was used, and the optical properties were averaged across the two layers. Simulation results were evaluated by plotting estimated versus reference SaO_2_ for both algorithms, while for the sheep experiments, time traces of fetal SpO_2_ estimates and reference SaO_2_ were compared.

## 3 Results

### 3.1 Simulation results

#### 3.1.1 *Simulated* Δ*OD_fetus_ across SD distances and wavelegnths*

**Figure 5.**
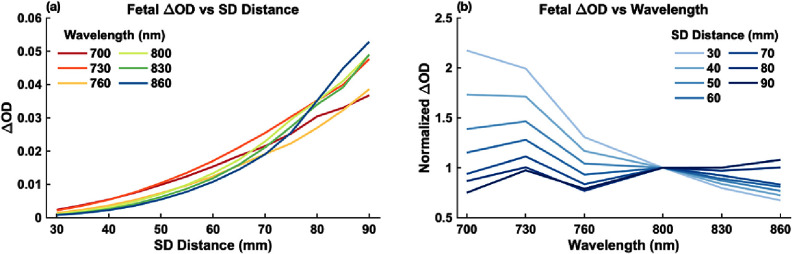
Representative ΔOD_fetus_ across SD distances and wavelengths. (a) Each curve corresponds to a specific wavelength, illustrating how the magnitude of fetal ΔOD increases as SD increases from 30 to 90 mm. (b) Each curve corresponds to a specific SD distance, illustrating how the spectral shape of ΔOD_fetus_ changes as SD increases from 30 to 90 mm.

Figure 5 presents an example of simulated ΔOD_fetus_ at a fetal depth of 20 mm across different SD distances and wavelengths. In figure 5(a), the amplitude of the ΔOD_fetus_ increases with longer SD distances, as expected due to enhanced sensitivity to deeper tissues. However, the signals become noisier at longer SD distances, reflecting increased photon attenuation and inherent Monte Carlo simulation noise. In figure 5(b), the spectral shapes of ΔOD_fetus_ change with increasing SD distance, indicating that measured signals progressively reflect a stronger fetal component compared to maternal tissue. These findings confirm the necessity of multi-layer modeling to correctly attribute changes in the measured signal to fetal SaO_2_.

#### 3.1.2 MAE analysis

**Figure 6.**
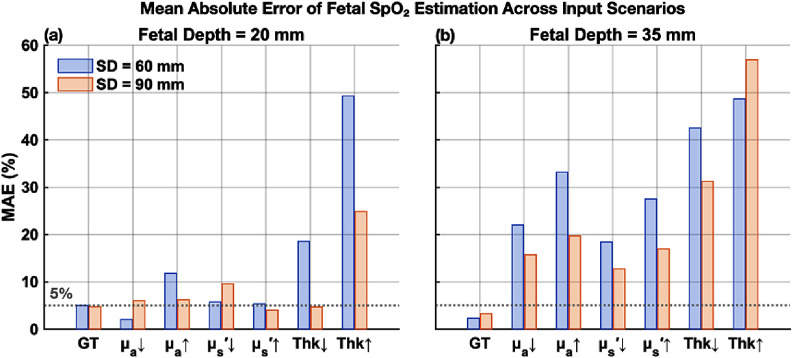
Mean absolute error (MAE) of fetal SpO_2_ estimation under input perturbations in ${\mu _{\text{a}}}$, $\mu _{\text{s}}^{\prime}$, and maternal-fetal thickness (Thk). ‘GT’ denotes the ground-truth case with accurate inputs. Results are shown for fetal depths of 20 mm (a) and 35 mm (b), with SD distances of 60 mm (blue) and 90 mm (orange). MAE = 5% is indicated by the gray dashed line.

The MAE of fetal SpO_2_ estimates under various input perturbations is summarized in figure 6. In the ideal case (GT input), the algorithm produced consistent low MAE values (<5%, gray dashed line) at both fetal depths (20 and 35 mm) and both SD distances (60 and 90 mm), demonstrating high accuracy under optimal conditions.

At the shallower fetal depth of 20 mm, perturbations in ${\mu _{\text{a}}}$ and $\mu _{\text{s}}^{\prime}$ inputs had relatively small effects on accuracy, with MAE typically increasing by less than or around 5%. At the greater depth of 35 mm, however, the impact of input perturbations became significant. With ±20% perturbations in ${\mu _{\text{a}}}$ and $\mu _{\text{s}}^{\prime}$, MAE values went up to ∼15%–30% from GT, indicating a clear increase in sensitivity to input uncertainties at greater fetal depth.

Errors in layer thickness had the greatest impact on fetal SpO_2_ accuracy, especially at 35 mm fetal depth. A 20% overestimation of fetal layer thickness led to extremely high MAE (>50%), highlighting that precise fetal depth measurement (e.g. via ultrasound) is critical for accurate fetal oximetry, particularly at greater depths.

Comparing the two SD distances (60 versus 90 mm), the longer SD distance generally showed slightly lower MAEs, particularly at 35 mm fetal depth, suggesting improved depth sensitivity and robustness at longer SD separations. However, differences between SD distances were modest at the shallower depth (20 mm).

#### 3.1.3 Error distribution analysis

**Figure 7.**
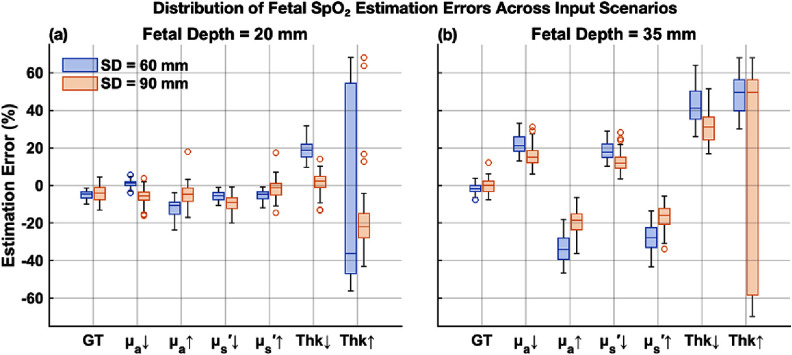
Distribution of fetal SpO_2_ estimation errors under the same input perturbations as in figure 6. Each boxplot shows the spread of individual-subject errors for a given condition: central lines denote medians, boxes indicate interquartile ranges, and whiskers represent data ranges excluding outliers. Results are shown for fetal depths of 20 mm (a) and 35 mm (b), with SD distances of 60 mm (blue) and 90 mm (orange).

Boxplots in figure 7 further illustrate the distribution and bias of estimation errors for fetal SpO_2_ across virtual subjects. In the ideal GT condition, errors were closely clustered around zero with narrow distributions, confirming robust baseline accuracy of the algorithm.

For optical property perturbations at the shallow fetal depth (20 mm), error distributions remained relatively close to zero, narrow, and symmetrical. At the deeper fetal depth (35 mm), however, error distributions became wider, reflecting increased uncertainty and reduced robustness to input errors. Clear directional biases were observed: underestimating ${\mu _{\text{a}}}$ and $\mu _{\text{s}}^{\prime}$ resulted in systematically overestimated SpO_2_, whereas overestimations led to underestimated SpO_2_.

**Figure 8.**
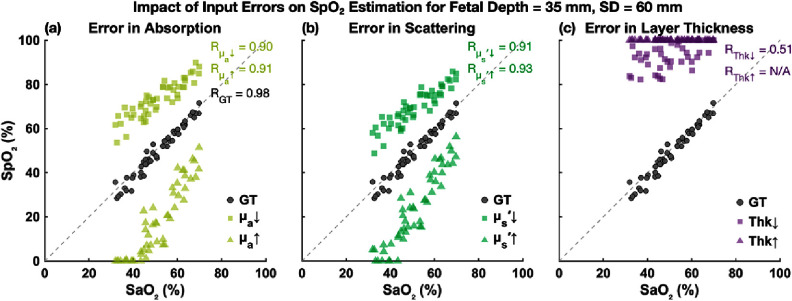
Scatter plot of estimated fetal SpO_2_ versus ground truth SaO_2_ at 35 mm fetal depth and 60 mm SD distance. Squares and triangles represent under and overestimation of input parameters, respectively.

**Figure 9.**
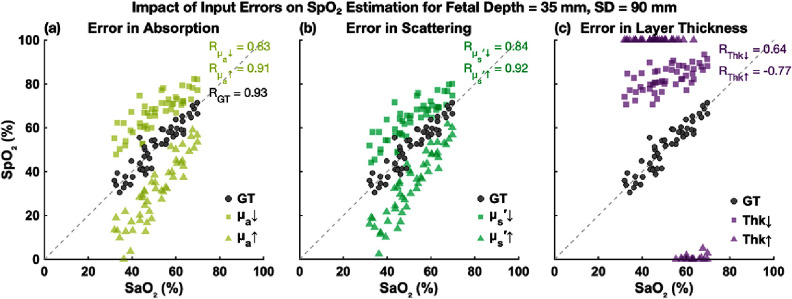
Scatter plot of estimated fetal SpO_2_ versus ground truth SaO_2_ at 35 mm fetal depth and 90 mm SD distance. Squares and triangles represent under and overestimation of input parameters, respectively.

Layer thickness perturbations introduced significant biases. Underestimating fetal depth resulted in consistent positive biases, whereas a 20% overestimation led to severe physiologically implausible SpO_2_ predictions (see scatter plots in figures 8 and 9) due to algorithm failure.

These findings emphasize that while optical property inaccuracies are important, precise fetal depth measurement is more crucial for the algorithm.

#### 3.1.4 Scatter plots of fetal SpO_2_ estimation

Scatter plots in figures 8 and 9 show individual estimates of fetal SpO_2_ versus true SaO_2_ at 35 mm fetal depth for 60 mm and 90 mm SD distances, respectively.

In the GT input scenario, estimated SpO_2_ values closely align with the unity line, with excellent correlation (*R* = 0.98 at 60 mm, *R* = 0.93 at 90 mm). Underestimation of optical properties caused clear upward shifts (SpO_2_ overestimation), while overestimation resulted in downward shifts (SpO_2_ underestimation), consistent with error distribution analysis. A similar upward shift occurred with underestimated fetal depth. However, the 20% overestimation in fetal depth (42 mm instead of 35 mm) led to significant algorithm failures, with SpO_2_ estimates saturating at 100% at 60 mm SD distance, making correlation values meaningless. At 90 mm SD, the estimates remained physiologically implausible with 100% and some 0% SpO_2_ estimations.

Correlation coefficients demonstrate that optical property errors still allowed moderate-to-high correlations, especially at 90 mm SD, but the precise fetal depth estimation is important.

### 3.2 In vivo sheep experiment results

#### 3.2.1 Δ*OD extraction from NIRS measurement*

To assess algorithm performance under *in vivo* conditions, we applied the multi-layer self-calibrated approach to NIRS data from a representative sheep experiment. Because clear fetal pulsations are difficult to detect in transabdominal fetal pulse oximetry, we selected a recording in which the fetal cardiac signal was distinctly visible in the spectrogram, providing a high-quality test case for evaluating the algorithm’s ability to track fetal SpO_2_ changes during controlled hypoxia.

**Figure 10.**
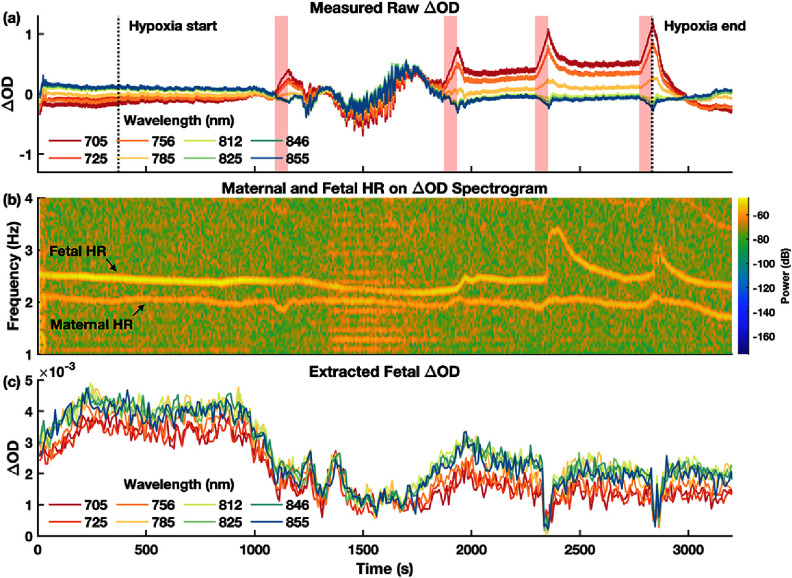
Example ΔOD_fetus_ extraction from NIRS measurement. (a) Raw ΔOD measured at 55 mm SD distance across all wavelengths. Vertical dashed lines indicate hypoxia onset and recovery of maternal hypoxia, and shaded regions denote 60 s ventilator holds. (b) Spectrogram from 812 nm wavelength, showing distinct maternal and fetal HR. (c) Isolated fetal component, ΔOD_fetus_, extracted at the identified fetal HR across all wavelengths.

Figure 10(a) shows the raw ΔOD_measured_(*t*) at a 55 mm SD distance across all wavelengths. The onset (initial reduction of maternal FiO_2_ from 50% to 37%) and end of hypoxia (increase of FiO_2_ to 100% for recovery) are indicated by the vertical dashed lines. Four 60 s ventilator holds are marked by the shaded regions. At the onset of hypoxia, the ΔOD signals at all wavelengths remained relatively stable. Each subsequent ventilator hold, however, induced clear changes in ΔOD with distinct wavelength-dependent patterns—the shorter wavelengths (705–756 nm) exhibited peaks at the end of apnea, while the longer ones (846–855 nm) showed corresponding troughs. This change in ΔOD spectral shape is consistent with the expected hemoglobin absorption spectrum under reduced oxygenation, indicating that both maternal and fetal tissues were undergoing desaturation. Following the end of hypoxia, ΔOD at all wavelengths gradually returned to baseline patterns.

The spectrogram corresponded to measurement at 812 nm wavelength is shown in figure 10(b). It is zoomed into 1–4 Hz frequency range to highlight distinct maternal and fetal HR components. We can see that fetal HR showed minimal change during the first ventilator hold, likely due to adequate placental oxygen reserve. In subsequent holds, however, clear tachycardia can be observed, indicating fetal response to hypoxemia. In contrast, maternal HR remained comparatively stable, likely due to greater physiological reserve.

**Figure 11.**
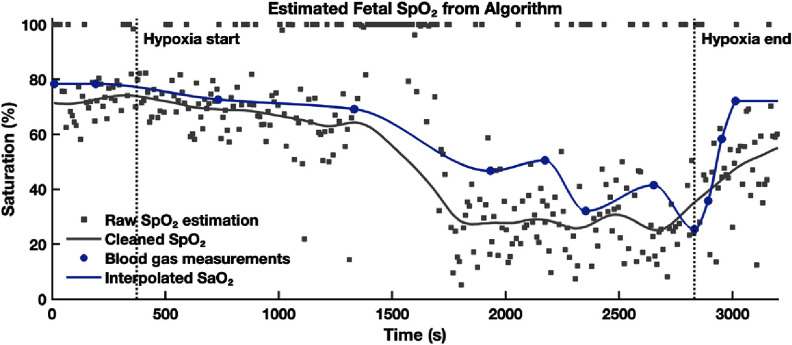
Estimated fetal SpO_2_ time trace from the multi-layer self-calibrated algorithm applied to ΔOD_fetus_. Raw SpO_2_ estimates (gray squares) and smoothed SpO_2_ (gray solid line) are shown alongside ground truth fetal SaO_2_ from blood gas analysis (blue circles) and its interpolated profile (blue line). Vertical dashed lines indicate hypoxia onset and recovery of maternal hypoxia.

Figure 10(c) shows the isolated fetal HR component (ΔOD_fetus_(*t*)) extracted using the time-frequency ridge method across all wavelengths. Despite relatively clear separation of maternal and fetal pulsations as shown in figure 10(b), the signals contained substantial noise. For example, around 1500 s, broadband interference was evident across frequencies, as also visible in figures 10(a) and (b). This artifact led to transient breakdown of fetal SpO_2_ estimation by the self-calibrated algorithm, as illustrated in figure 11. In addition, during the final two ventilator holds, the pronounced fetal tachycardia likely reduced the accuracy in ΔOD_fetus_(*t*) extraction using the time-frequency ridge method.

#### 3.2.2 Estimating fetal SpO_2_ from sheep data

The extracted ΔOD_fetus_(*t*) was processed using the multi-layer self-calibrated algorithm to produce a continuous fetal SpO_2_ time trace. Figure 11 shows the raw SpO_2_ estimates (gray squares), which exhibited recognizable physiological trends but also with substantial noise. Following the cleaning and smoothing procedure described in section 2.4.3, the refined SpO_2_ trace (gray solid line) is plotted alongside GT fetal SaO_2_ from arterial blood gas analysis (blue circles) and its interpolated profile (blue line).

From the estimated fetal SpO_2_, we can observe clear physiological correspondence: baseline fetal SpO_2_ was ∼80%, gradually decreasing during the induced hypoxia and reaching ∼25% toward the end of the hypoxic period. After the hypoxia ended, SpO_2_ recovered to ∼75%. Although the algorithm had some overall underestimation and showed a breakdown around 1500 s, likely due to broadband noise interference observed across frequencies as seen in figure 10, this agreement supports the physiological validity of the algorithm’s output.

**Figure 12.**
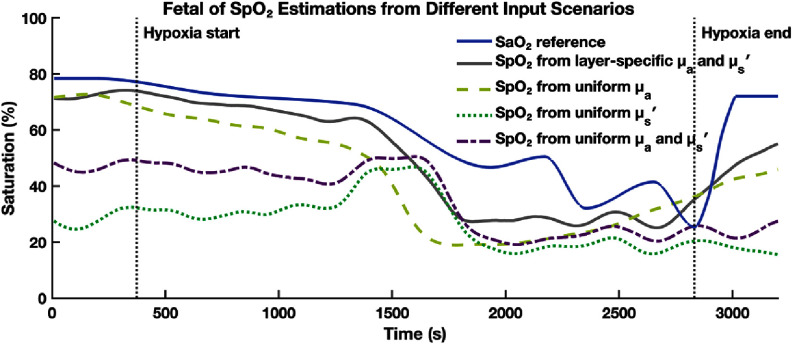
Estimated fetal SpO_2_ from different input scenarios: layer-specific optical properties (gray), uniform absorption (light green), uniform scattering (dark green), and uniform absorption and scattering (purple). All traces are smoothed and shown alongside the ground truth SaO_2_ (blue). Vertical dashed lines indicate hypoxia onset and recovery of maternal hypoxia.

We then tested algorithm under three perturbed optical property assumptions: (1) uniform absorption across maternal and fetal layers but with layer-specific scattering, (2) uniform scattering but with layer-specific absorption, and (3) uniform absorption and scattering for both layers. As shown in figure 12, all perturbed conditions produced reduced accuracy, but we could still see some broad oxygenation trend. Especially in the uniform absorption case (case1, light green), the overall trajectory was preserved. In cases 2 and 3, we can see the estimated fetal SpO_2_ decreases from baseline to the hypoxic nadir, but the expected high-low-high oxygenation trends were not apparent.

**Figure 13.**
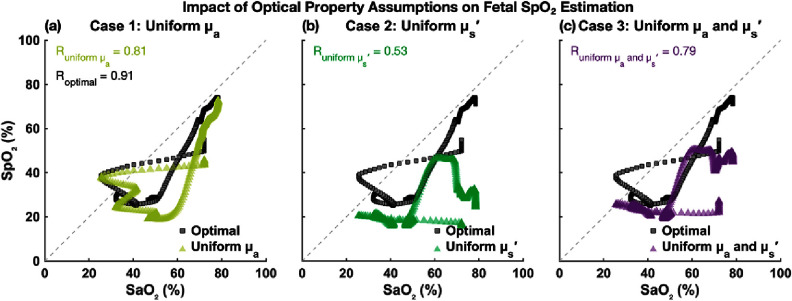
Scatter plots comparing fetal SpO_2_ estimations to ground truth SaO_2_ for different input scenarios. (a) Assuming uniform absorption across layers (light green). (b) Assuming uniform scattering (dark green). (c) Assuming both uniform absorption and scattering (purple). Each panel shows Pearson correlation coefficient (*R*). The optimized case with layer-specific optical properties (gray square) yielded the highest correlation and lowest error.

Figures 13(a)–(c) presents the scatter plots of smoothed SpO_2_ versus interpolated SaO_2_ for the optimized case with layer specific optical properties and each of the three perturbed cases. In the optimized case (gray squares), data points generally follow the unity line with some underestimation and spread, resulting in a Pearson correlation coefficient *R* = 0.91 and MAE = 10.3%. All three perturbed cases showed greater deviation from the unity line and higher errors: case 1 (*R* = 0.81, MAE = 16.5%), case 2 (*R* = 0.53, MAE = 33.0%), and case 3 (*R* = 0.79, MAE = 24.2%). Among the perturbed scenarios, case 1 (uniform ${\mu _{\text{a}}}$) retained the strongest agreement with the reference SaO_2_, suggesting that scattering contrast might play a more critical role in accurate fetal SpO_2_ estimation.

### 3.3 Comparison between single-layer and multi-layer self-calibrated algorithms

To evaluate the improvement achieved by the multi-layer self-calibrated algorithm over the single-layer version, both approaches were applied to simulated and *in vivo* datasets.

**Figure 14.**
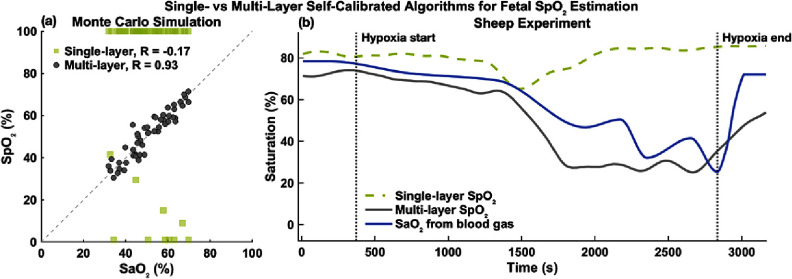
Comparison of single-layer and multi-layer self-calibrated algorithms. (a) Monte Carlo simulation results for a representative case with a fetal depth of 35 mm and a SD distance of 90 mm. Multi-layer (gray circles) and single-layer (light green squares) results are shown. (b) *In vivo* results from the sheep experiment. Fetal SpO_2_ from both the multi-layer (gray solid line) and single-layer (light green dashed line) algorithms are smoothed and plotted alongside the reference SaO_2_ (blue solid line). Vertical dashed lines indicate the onset and recovery of maternal hypoxia.

Figure 14(a) presents result from the Monte Carlo simulations for a fetal depth of 35 mm and a SD distance of 90 mm. The multi-layer model (gray circles) showed a strong correlation with the GT fetal SaO_2_ (*R* = 0.98, MAE = 3.2%). The single-layer model (light green squares), however, failed to produce meaningful estimates (*R* = − 0.17, MAE = 48.6%). The poor performance of the single-layer model arises from its inability to account for differential photon penetration through maternal and fetal layers, which have different optical properties and oxygen saturations, leading to algorithm breakdown.

Figure 14(b) presents result from the sheep experiment. The multi-layer algorithm (gray solid line) tracked the overall fetal desaturation and recovery dynamics induced by maternal hypoxia, closely corresponded to the reference SaO_2_ (blue solid line) with *R* = 0.91 and MAE = 10.3%. In contrast, the single-layer algorithm (light green dashed line) again failed to capture the meaningful physiological trend (*R* = − 0.27, MAE = 20.7%).

Overall, these findings demonstrate that the multi-layer implementation substantially enhances physiological fidelity and quantitative accuracy in fetal SpO_2_ estimation, whereas the single-layer approach is insufficient for resolving layered tissue contributions.

## 4 Discussion

In this study, we present a multi-layer self-calibrated algorithm for estimating fetal arterial oxygen saturation from non-invasive transabdominal NIRS measurements. By incorporating model of photon partial pathlengths for layered maternal-fetal geometry, our approach addresses two limitations of conventional pulse oximetry method: the implicit assumption of tissue homogeneity and reliance on empirical calibrations derived from healthy adults.

Monte Carlo simulations provided a controlled environment to examine how fetal depth, SD distance, and input optical properties affect estimation accuracy. Notably, when the appropriate input parameters (layer thickness and optical properties) are used, shorter separation (60 mm) achieved accuracy comparable to longer separation (90 mm) even at fetal depths of 35 mm (figures 8 and 9). This suggests that sensitivity losses at shorter SD could be compensated if layer contributions are modeled correctly analytically and opens a path to simpler hardware without sacrificing deep-layer sensitivity. Perturbation analyzes further showed that errors in input parameters introduce directional biases (underestimated ${\mu _{\text{a}}}$ or $\mu _{\text{s}}^{\prime}$ caused overestimated fetal SpO_2_, and vice versa), with thickness errors producing the largest degradations. These observations motivate complementary hardware modality (e.g. ultrasound) for layer thickness and advanced diffuse optical techniques to inform individual- and layer-specific optical properties.

Sheep experiment served as a proof-of-concept demonstration under *in vivo* condition. The algorithm tracked expected fetal oxygenation dynamics: a decline from ∼80% at baseline to ∼25% at hypoxia nadir, followed by recovery. However, several limitations should be acknowledged. First, fetal hypoxia was induced by maternal desaturation, meaning concurrent maternal and fetal saturation changes may confound estimates. Umbilical cord compression would more specifically perturb fetal oxygenation, but it carries higher risk of fetal distress [33]. Second, layer properties were not measured directly but selected by grid search from physiologically plausible values to match baseline fetal SpO_2_, which is not applicable for clinical setting. Also, while simulations suggest robustness to maternal-layer variability given accurate inputs, translation to uncontrolled clinical settings will require independent, non-invasive optical property estimation. Third, the analytical partial-pathlength model we used assumes flat, stratified layers, but abdominal curvature and heterogeneity in both sheep and humans violate this assumption. Future work should therefore consider anatomical priors, curvature corrections, and model adaptations [51–53]. Lastly, the *in vivo* validation was performed in a single animal, which limits the statistical strength and generalizability of the findings. Expanding the sample size in future studies will be essential to assess inter-subject variability and to confirm reproducibility under varying physiological and anatomical conditions.

To illustrate the advantages of the multi-layer approach, we compared its performance with the previous single-layer model using the same simulation and *in vivo* datasets. The single-layer model consistently produced physiologically implausible fetal SpO_2_ estimates and exhibited larger errors in both simulated and experimental data. These findings demonstrate that neglecting layer heterogeneity introduces significant bias and is insufficient for the self-calibrated algorithm to resolve oxygenation in thick, layered maternal and fetal tissues.

For clinical context, there is currently no transabdominal fetal pulse oximeter in routine clinical use. Historically, the only device cleared for clinical use was a transvaginal fetal oximeter (Nellcor N400), which required direct placement on the fetal head and was discontinued because of limited applicability [10, 11, 54]. Compared to historical benchmarks, Luttkus *et al* reported a median absolute difference of 5.2% (95% CI 2.5%–10.3%) versus fetal scalp SaO_2_ across ∼10%–70% saturation, with signed errors from −21% to +36% and a tendency to overestimate [55]. In our sheep study, we calculated the median absolute difference to be 7.2% (95% CI 6.6%–7.9%), with signed differences from −23% to +13%. While our median error is modestly larger, the interval is narrower and the bias smaller in magnitude, potentially indicating more consistent error behavior with a slight underestimation tendency (figure 11), which is related to the input parameter selection bias observed in simulation. For a more recent transabdominal approach by Fong *et al* [56], they first extracted fetal signals by subtracting a short separation ‘maternal’ channel from a longer ‘maternal + fetal’ channel. Then, in a sheep hypoxia experiment, they performed calibration on a subset of the same data used for evaluation and reported root mean square error RMSE = 6.4% and Pearson *R*= 0.93. Our *in vivo* results (RMSE = 12.2%, *R* = 0.91) show similar correlation, but higher RMSE. However, long-short channel subtraction approach does not explicitly model layer-specific sensitivity or partial pathlengths, and non-independent calibration/testing likely biases RMSE downward. Using transabdominal measurements, Qian *et al* [57] introduced a machine-learning approach for binary detection of instantaneous fetal hypoxemia (defined as fetal SaO_2_ < 30%), achieving ∼88% accuracy in a controlled sheep model. This work demonstrates the potential of data-driven fusion of multi-detector signals for classification, which could complement analytical frameworks such as ours. However, its reliance on binary thresholding (normoxia versus hypoxemia) and the constrained experimental conditions represented in the training dataset may limit physiological interpretability and generalization to broader populations.

A key translational challenge of our approach is to obtain accurate, layer-specific thicknesses and optical properties, as these parameters jointly determine photon pathlengths. Since different combinations of layer thickness and optical properties can yield similar reflectance spectra [47], and the self-calibrated algorithm relies on the spectral shape of ΔOD_fetus_, accurate characterization of these parameters is essential for robust SpO_2_ estimation.

For layer thickness and fetal depth estimation in human applications, this limitation may be mitigated by integrating auxiliary imaging modalities such as ultrasound for real-time depth tracking or by constructing individualized anatomical priors from MRI-derived abdominal geometry. Although focused on oncology, Geldof *et al* [58] demonstrated that diffuse reflectance spectroscopy alone can recover layer thickness and classify tissue types with sub-millimeter accuracy in a two-layer model (<15 mm top layer). This suggests potential for optical approaches to retrieve fetal depth in transabdominal fetal pulse oximetry.

For optical property estimation, future work could combine frequency-domain or time-domain NIRS with analytical or data-driven inversion models to jointly estimate absorption and scattering coefficients *in vivo*. Forward models coupled with inversion frameworks have shown promise in recovering layer-specific optical properties [46, 47, 59–62]. Recent advances including machine learning methods [63, 64], deep learning architectures such as CNNs [65] and U-Nets [66], and Bayesian methods [46, 67], have demonstrated the feasibility of learning-based and probabilistic reconstruction of spatially varying optical properties. Extending these methods to transabdominal measurements, particularly with high-density multi-distance, multi-wavelength SD configurations, may enable simultaneous estimation of fetal depth and layer optical properties.

In addition, for human applications where the fetal depth is substantially greater than in the present animal model, enhanced depth-sensitive techniques such as interferometric NIRS [68] or dual-slope approaches [69, 70] could improve sensitivity to deeper fetal tissues. Wavelength selection and SD placement are also critical, as they determine both measurement depth and chromophore sensitivity [39, 71]. Optimizing wavelength combinations to emphasize low-oxygen hemoglobin features [12, 16] and account for potential melanin influence [72, 73] may further improve algorithm performance.

Separating weak fetal pulsations from dominant maternal signals is also critical. In this study, fetal HR was tracked using spectrogram analysis and ridge detection. However, in clinical settings, maternal motion, respiration, uterine contractions, and other factors will likely introduce additional interference, necessitating advanced signal-processing approaches. Future work should explore methods such as adaptive filtering with physiological [74], time-frequency decomposition [75], and others [76, 77], which have already shown promise. Auxiliary sensors, such as fetal ECG, could further improve fetal HR tracking [7, 78, 79].

The current MATLAB implementation computes each SpO_2_ estimate in ∼2 s per frame on a MacBook Air (M2, 16 GB) using parallel processing, which is not yet suitable for real-time monitoring. Computation speed can be substantially improved through vectorization, pre-computation of pathlength terms, and migration to compiled languages such as Julia or C++, where solver for photon diffusion in layered media has achieved high accuracy and sub-millisecond fluence calculations [29]. Recent progress in embedded optical hardware further supports the feasibility of real-time deployment. Recent handheld frequency-domain NIRS device integrate FPGA-based inverse modeling and on-board digital signal processing, achieving real-time (>10 Hz) 2D mapping of optical properties [62]. Similarly, handheld diffuse optical tomography probe combining high-speed acquisition with transformer-based neural reconstruction are being developed [80]. For transabdominal fetal monitoring, future designs could adopt a wireless multimodal sensor architecture that integrates optical, inertial, and electrophysiological sensing for simultaneous maternal-fetal assessment, as demonstrated by recent wearable prototypes for placental oxygenation monitoring [81]. With computation optimization and hardware miniaturization, real-time fetal SpO_2_ feedback in a wearable format appears technically achievable.

In conclusion, the proposed multi-layer self-calibrated algorithm demonstrated feasibility in both simulations and *in vivo* sheep experiments for tracking fetal oxygenation trends from non-invasive transabdominal NIRS measurements. While its performance depends on accurate optical property and layer thickness inputs, it remains informative under moderate parameter uncertainties. These findings motivate further development of optical property estimation methods, signal and hardware optimization strategies, and validation in human studies. Our long-term goal is to provide reliable fetal oxygenation monitoring during labor, ultimately improving maternal and fetal outcomes.

## Data Availability

The data that support the findings of this study are openly available at the following URL/DOI: https://github.com/jingyiwu-biophotonics/multi-layer-self-calibrated-fetal-pulse-oximetry. Experimental data from the sheep studies are not publicly available due to ownership restrictions.
